# Human immunodeficiency virus transmission from a 24-year-old woman to her 78-year-old grandmother: a case report

**DOI:** 10.1186/s13256-021-02918-y

**Published:** 2021-07-10

**Authors:** Pedro Pallangyo, Jalack Millinga, Happy Swai, Naairah R. Hemed, Zabela Mkojera, Silvia Mosha, Marcelina Granima, Smita Bhalia, Yona Gandye, Mohamed Janabi

**Affiliations:** 1 Department of Research Training, Jakaya Kikwete Cardiac Institute, P.O Box 65141, Dar es Salaam, Tanzania; 2 Department of Nursing, Jakaya Kikwete Cardiac Institute, P.O Box 65141, Dar es Salaam, Tanzania; 3 Department of Adult Cardiology, Jakaya Kikwete Cardiac Institute, P.O Box 65141, Dar es Salaam, Tanzania

**Keywords:** HIV infection, Unusual HIV transmission, Rare HIV transmission, Accidental HIV transmission, Case report

## Abstract

**Introduction:**

Since its debut recognition in 1981, human immunodeficiency virus/acquired immunodeficiency syndrome has affected over 77 million people and has resulted in premature cessation of 35.4 million lives worldwide. Commonly, human immunodeficiency virus is transmitted by sexual contact across mucosal surfaces, by sharing of injecting equipment, through contaminated blood transfusions, and by maternal–infant exposure. Nevertheless, accidental transmission incidences involving family members are rare but possible.

**Case presentation:**

A 78-year-old woman of African descent from Mtwara Region south of Tanzania was referred to us for further evaluation and treatment. She is 30 years postmenopausal and has a 35-year history of hypertension. Her last attendance to our institute was 11 months prior the index visit and she tested negative for human immunodeficiency virus. She came with complaints of weight loss, recurrent fevers, and cough. Her hematological tests revealed leukopenia with lymphocytosis, together with a normocytic normochromic anemia. Enzyme-linked immunosorbent assay for human immunodeficiency virus was positive, and she had a CD4 count of 177 cells/µL. We went back to history taking to identify the potential source of infection. We were informed that for the past 6 months, the 78-year-old lady has been living with her unwell 24-year-old granddaughter who has been divorced. The granddaughter had a history of recurrent fevers, significant weight loss, and a suppurative skin condition. As a way to show love and care, the old lady was puncturing the suppurative lesions with bare hands; then she would suck them to clear away the discharge. We requested to see the young lady, and she tested positive for human immunodeficiency virus. Both were started on tenofovir/lamivudine/dolutegravir combination plus cotrimoxazole 960 mg. The family was in total disarray following these findings. The patient was discharged through infectious diseases department and died of *Pneumocystis jirovecii* pneumonia 12 weeks later.

**Conclusions:**

Certain sociocultural norms that are believed to express love, care, and togetherness in developing rural communities, particularly Sub-Saharan Africa, have a potential of spreading human immunodeficiency virus, thus warranting prompt transformation.

## Introduction

Since its debut recognition in 1981, human immunodeficiency virus/acquired immunodeficiency syndrome (HIV/AIDS) has continued to devastate families and communities all over the globe. Thus far, HIV has affected over 77 million people and has resulted in premature cessation of 35.4 million lives worldwide [1, [2]. Harboring over two-thirds of global HIV infections and fatalities, the World Health Organization (WHO) African region remains the hardest-hit region [3]. Owing to the combination of highly active antiretroviral therapy (HAART), a remarkable transformation from a rapidly fatal to a chronic manageable condition with dramatic survival prolongation has been witnessed.

Commonly, HIV is transmitted by sexual contact across mucosal surfaces, by sharing of injecting equipment, through contaminated blood transfusions, and by maternal–infant exposure [4]. Nonetheless, numerous unusual transmission incidences such as father to son [5], from one child to another [6], between two adolescent brothers [7], from child to mother [8], between two females [9], due to human bite [10], from a traditional healer to client [11], and via fights [12–14] have been documented in the literature. By virtue of HIV’s dynamic nature and multiple transmission modalities, it is crucial to establish the likely route of infection if the pandemic is to be fully understood. We report an accidental yet rare case of HIV transmission from a 24-year-old woman of African descent to her 78-year-old grandmother.

## Case presentation

A 78-year-old woman of African descent from Mtwara Region south of Tanzania was referred to us for further evaluation and treatment. She was a retired peasant and mother to nine children. She was 30 years postmenopausal and had a 35-year history of hypertension on telmisartan 80 mg once daily, clopidogrel 75 mg once daily, and metoprolol 50 mg once daily. There was a negative history of tobacco use; however, she had been consuming local brewery since her youth. Her last visit to our institute was 11 months ago in which she underwent coronary angiography (CAG) for evaluation of coronary artery disease. Her CAG findings revealed nonobstructive coronary artery disease, and her medications were optimized. As per protocol, prior to CAG, she underwent several hematological, biochemical, and serological tests including HIV test, which all revealed essentially normal findings.

She presented with a 2-month history of general body malaise, weight loss, recurrent fevers, and cough. Physical examination was uneventful except for a wasted body habitus. She had stable vitals, that is, blood pressure 132/84 mmHg, pulse 68 beats/minute, respiratory rate 29 breaths/minute, temperature 37.2°C, and oxygen saturation 97%. Renal function, liver function, and chemistry panel revealed normal findings; however, hematological tests revealed leukopenia (WBC 2100 × 10^9^/L) with lymphocytosis, and normocytic normochromic anemia (Hb 7.2 g/dL). Such findings together with the clinical picture prompted us to do a rapid HIV test, which was reactive to both SD Bioline and Uni-Gold tests. To clear our doubts, we performed enzyme-linked immunosorbent assay (ELISA) for HIV, and she was reactive. She had a CD4 count of 177 cells/µL. Such results surprised us all mainly due to the fact that our patient has been sexually inactive for over 30 years and has not been transfused all her life. We knew something somehow somewhere was missing, and we had to go back to history taking.

This time, we interviewed her daughter, and after several minutes of probing, a shocking revelation came out. We were informed that for the past 8 months, the 78-year old lady had been living with her unwell 24-year-old granddaughter who has been divorced. The granddaughter had a history of recurrent fevers, significant weight loss, and a suppurative skin condition. As a way to show love and care, the old lady was puncturing the suppurative lesions with her bare hands; then she would suck them to clear away the discharge. The informant denied any knowledge of the HIV serostatus of the 24-year-old lady. We requested to see the young lady, and she was brought to us 3 days later. She looked frail, emaciated, and had multiple generalized skin lesions. She denied using antiretroviral drugs (ARVs) or knowing her HIV status. After precounseling, she agreed to test for HIV, and she was infected. She had a CD4 count of 144 cells/µL. Both were started on tenofovir/lamivudine/dolutegravir (TLD) combination plus cotrimoxazole 960 mg. The family was in total disarray following these findings. The patient was discharged through infectious diseases department and died of *Pneumocystis jirovecii* pneumonia 12 weeks later.

## Discussion

In the battle against HIV/AIDS, it is essential to acknowledge all routes of transmission if the pandemic is to be fully understood and well controlled. This present case report from a resource-limited setting underscores the HIV dynamicity in its transmission and raises concerns over certain social cultural practices in impoverished societies.

The HIV and AIDS pandemic continues to threaten the global public health and remains a stumbling block to human development. In the last decade, the world has witnessed an 18% decline in new HIV infections [2]. Encouragingly, the new infections have gone down by 30% in Sub-Saharan Africa (SSA) which is the most affected region [2]. However, despite this global downward trend, some regions are experiencing a rapid rise in new infections and are struggling to expand treatment [15]. The joint United Nations programme on HIV/AIDS (UNAIDS) launched the 90–90–90 (that is, 90% of infected individuals to know their status, 90% to receive sustained HAART, and 90% to achieve viral suppression by 2020) targets in 2014 as a strategy to hasten progress towards eliminating AIDS by 2030 [16].

Today, our scientific understanding of HIV transmission, treatment, and prevention has changed the disease landscape remarkably. Significant challenges, however, persist as innumerable social, cultural, economic, political, and legal barriers complicate the efforts in addressing this pandemic [17, 18]. Although HIV/AIDS has been present for nearly four decades, there is still a widespread misunderstanding about how it is spread, consequences of infection, and prevention methods, especially in SSA [19]. In such communities, women of rural areas, those with low education level, and women dependent on men financially are reported to be the most vulnerable groups [19]. Cultural norms continue to be linked with the spread of HIV/AIDS, particularly in SSA [11]. In the present case, extensive history taking coupled with documented past medical history found no evidence of any form of sexual contact or blood transfusion that could explain the HIV seropositive status of the 78-year-old. Nevertheless, based on the information gathered, we are convinced that the old lady acquired the infection from her granddaughter through the extraordinary nursing of the suppurative skin lesions.

## Conclusions

Despite its infrequency, intrafamilial transmission of HIV should be considered as a potential mode of infection, especially when an anticipated source is not apparent. Certain sociocultural norms that are believed to express love, care, and togetherness in developing rural communities potentially spread HIV, thus demanding prompt transformation.

## Data Availability

Not applicable.

## Abbreviations

AIDS: Acquired immune deficiency syndrome
ARVs: Antiretroviral drugs
BP: Blood pressure
CAG: Coronary angiogram
ELISA: Enzyme-linked immunosorbent assay
HAART: Highly active antiretroviral therapy
Hb: Hemoglobin
HIV: Human immunodeficiency virus
SSA: Sub-Saharan Africa
TLD: Tenofovir/lamivudine/dolutegravir ART combination
UNAIDS: The Joint United Nations Programme on HIV and AIDS
WBC: White blood cells
WHO: World Health Organization
